# Anterior Referencing versus Posterior Referencing in Primary Total Knee Arthroplasty: A Meta-Analysis of Randomized Controlled Trials

**DOI:** 10.3390/jcm12237453

**Published:** 2023-12-01

**Authors:** Filippo Familiari, Michele Mercurio, Francesco Napoleone, Olimpio Galasso, Ermes Giuzio, Roberto Simonetta, Michelangelo Palco, Nicholas N. DePhillipo, Giorgio Gasparini

**Affiliations:** 1 Department of Orthopaedic and Trauma Surgery, Magna Graecia University, 88100 Catanzaro, Italy; mercuriomi@gmail.com (M.M.); napoleous@gmail.com (F.N.); galasso@unicz.it (O.G.); gasparini@unicz.it (G.G.); 2 Research Center on Musculoskeletal Health, MusculoSkeletalHealth@UMG, Magna Graecia University, 88100 Catanzaro, Italy; 3 Division of Orthopaedic and Trauma Surgery, Villa del Sole Clinic, 88100 Catanzaro, Italy; ermes.giuzio@tiscali.it (E.G.); dr.simonetta@gmail.com (R.S.); michelangelo.palco@gmail.com (M.P.); 4 Department of Orthopaedic Surgery, University of Pennsylvania, Philadelphia, PA 19104, USA; nicholas.dephillipo@pennmedicine.upenn.edu

**Keywords:** systematic review, meta-analysis, anterior referencing, randomized controlled trials, posterior referencing, total knee arthroplasty, clinical outcome, radiographic outcome

## Abstract

(1) Background: The purpose of this study was to perform a systematic review and meta-analysis of studies comparing clinical and radiographic outcomes between anterior referencing (AR) and posterior referencing (PR) systems in total knee arthroplasty (TKA). (2) Methods: This study followed the Preferred Reporting Items for Systematic Reviews and Meta-Analyses (PRISMA) statement. PubMed, MEDLINE, Scopus, and Cochrane Central databases were searched in August 2022. Data extracted for quantitative analysis included the Knee Society Score (KSS), the Western Ontario and McMaster University (WOMAC) index, knee ROM, posterior condylar offset (PCO), and the posterior condylar offset ratio (PCOR). The methodological quality of the included studies was assessed using the Modified Newcastle–Ottawa Quality Assessment. Randomized controlled trials were assessed with version 2 of the risk of bias tool (RoB2), recommended by the Cochrane Collaboration. (3) Results: For the meta-analysis, five comparative studies met the eligibility criteria. There were 584 patients in all, 294 of whom had AR TKA and 290 of whom had PR TKA. Three studies with 181 and 179 cases in the AR and PR groups, respectively, had reported preoperative KSS. A statistically significant difference was found favoring the PR group. (*p* = 0.01). The same cases’ postoperative range of motion was documented, and a statistically significant difference was discovered in favor of the AR group. Postoperative PCO was described in four studies in 243 and 241 cases in the AR and PR TKA groups, respectively, and a statistically significant difference was found with a higher postoperative PCO in the PR group (*p* = 0.003). Postoperative PCOR was calculated in two studies in the same cases in the AR and PR TKA groups and a statistically significant difference was found with a higher postoperative PCOR in the PR group (*p* = 0.002). (4) Conclusion: Anterior referencing for TKA may result in improved knee ROM postoperatively, while posterior referencing may produce larger PCO and PCOR on postoperative imaging. However, no significant differences were noted in clinical outcomes between the AR and PR groups at final follow-up.

## 1. Introduction

As we delve into the expansive realm of Total Knee Arthroplasty (TKA), it becomes evident that the landscape of musculoskeletal healthcare is undergoing a transformative evolution. TKA has transcended its role as a routine orthopedic procedure to become a cornerstone in orthopedic practice, witnessing a substantial annual increase globally [1,2,3]. This surge in prevalence sparks curiosity and prompts a deeper exploration into the driving factors behind this trend, seeking to unravel the complex interplay of demographic shifts, technological advancements, and evolving patient expectations [1,2,3]. The growing acceptance and adoption of TKA underscore not only its clinical efficacy but also its profound impact on the lives of individuals grappling with the debilitating consequences of end-stage knee osteoarthritis (OA).

Renowned for its safety and efficacy, TKA emerges as a paramount method for addressing the multifaceted challenges posed by end-stage knee OA. It offers a beacon of hope and much-needed relief for patients navigating the tumultuous terrain of functional limitations, diminished quality of life, and incapacitating articular pain. The increasing prevalence of TKA attests to its transformative power in restoring not only joint function but also the overall well-being of individuals burdened by the burdensome impact of OA.

In this metamorphosis of musculoskeletal healthcare, the preservation of joint anatomy emerges as a pivotal factor, propelling TKA beyond a mere procedural intervention to the realm of meticulous artistry. The insertion of a prosthesis, when executed with precision, involves the meticulous reproduction of bone morphology, the maintenance of the joint line, and the restoration of native knee biomechanics. This multifaceted approach plays a vital role in delaying component wear and, by extension, facilitates potential revision surgeries [4,5]. The emphasis on joint preservation signals a paradigm shift in orthopedic surgery, transcending the traditional focus on symptomatic relief to a more holistic approach that considers the long-term implications for patient well-being.

Consequently, the trifecta of proper implant positioning, secure fixation [6], and accurate sizing assumes a central role in ensuring the overall success of the TKA procedure [7,8,9]. The pursuit of excellence in these technical aspects is not merely a reflection of surgical proficiency but, more importantly, underscores the unwavering commitment of orthopedic surgeons to achieving optimal patient outcomes. The dedication to precision goes beyond enhancing the immediate postoperative period; it aligns with broader goals aimed at improving the quality of life for patients over the long term.

In the intricate dance between anatomy and technology, two major referencing systems, Anterior Referencing (AR) and Posterior Referencing (PR), dominate the landscape of femoral component positioning and sizing. The alignment of the anteroposterior size of the femoral condyle with that of the component dictates the uniformity of bone resection, irrespective of the referencing method employed. However, the nuances become apparent when the femoral condyle’s anteroposterior size falls between available component sizes. Opting for AR results in more posterior bone resection, widening the flexion gap with smaller component sizes, potentially leading to anterior notch formation with larger sizes [10]. Conversely, PR may increase the risk of anterior clearance tightness and potential patellofemoral overstuffing with larger component sizes [10]. This intricate dance requires not only technical prowess but also a profound understanding of the patient’s unique anatomical characteristics.

Theoretical drawbacks associated with each referencing system can be mitigated through the application of meticulous surgical techniques, advanced surgical instruments, and the utilization of newly developed implants [11]. The commitment to overcoming theoretical challenges stands as a testament to the resilience of the orthopedic community in the face of evolving surgical landscapes. The integration of advanced technologies and innovative implants reflects a commitment to continuous improvement, ensuring that the field of orthopedics remains at the forefront of medical innovation.

The intricate kinematic behavior of the knee post-TKA, particularly concerning the range-of-motion (ROM), poses a significant challenge for prediction. Stiffness following TKA has been reported in up to 1.3% of cases [12], adding a layer of complexity to the postoperative phase. Factors influencing the final ROM encompass various surgical parameters, such as implant fixation technique [13], implant type, tibial slope, posterior condylar offset, and the Insall index, all of which impact the joint line’s level [14,15,16]. Conversely, preoperative factors like knee flexion, gender, body mass index (BMI), and psychological variables remain beyond the surgeon’s control [17,18,19]. This intricate dance between patient-specific factors and surgical techniques adds a layer of complexity to the personalized care offered in the field of orthopedics, emphasizing the need for tailored approaches to optimize outcomes.

Despite the considerable advancements in TKA, the impact of AR and PR on ROM remains a subject of ongoing debate. Understanding the interplay between these referencing systems and their effects on clinical and radiographic outcomes is essential for refining surgical practices and optimizing patient outcomes. This acknowledgment of the ongoing discourse emphasizes the dynamic nature of orthopedic research, wherein the pursuit of knowledge is as crucial as the application of existing evidence in clinical settings.

In light of these considerations, the primary objective of our study is to conduct a comprehensive systematic review and meta-analysis. This analysis will scrutinize existing studies, comparing the clinical and radiographic outcomes between AR and PR systems in TKA. Through this investigation, we aim to contribute valuable insights to the ongoing discourse surrounding optimal referencing systems in TKA, shedding light on potential avenues for improvement and innovation in this critical orthopedic procedure. The commitment to a systematic review and meta-analysis reflects the dedication of the orthopedic community to evidence-based practices and continuous improvement. This research endeavor not only aims to synthesize existing knowledge but also paves the way for future investigations.

## 2. Materials and Methods

This study followed the Preferred Reporting Items for Systematic Reviews and Meta-Analyses (PRISMA) statement. PubMed, MEDLINE, Scopus, and Cochrane Central databases were searched in August 2022. The terms “anterior”, “posterior”, “referencing”, “total”, “knee”, “arthroplasty”, “replacement”, “outcome”, and “results” were used in different combinations to retrieve relevant articles. Two authors (M.M. and F.N.) independently screened the titles and the abstracts to identify articles for inclusion, contacting a third senior author (F.F.) in case of major discrepancies. We looked for possible extra articles to include by screening the references lists of all included articles and our institution’s gray literature collection.

### 2.1. Inclusion Criteria and Study Selection

Inclusion criteria were applied during title, abstract, and full text screenings; these were defined as follows: (1) observational studies including case-control, cohort studies, and randomized controlled trials (RCT); (2) reporting comparative outcomes of anterior versus posterior referencing for TKA for the treatment of end-stage knee OA; (3) reporting of >10 surgically treated cases; and (4) articles written in English. Excluded from the analysis were other reviews, case reports, cadaveric or biomechanical studies, technical notes, editorials, letters to the editor, and expert opinions.

### 2.2. Data Extraction and Quality Assessment

Two authors (M.M. and F.N.) performed comprehensive data extraction from the included articles. The first author, journal name, year of publication, study design, patient demographics, type of referencing for femoral component, type of implant, follow-up period, and outcomes were recorded. Data extracted for quantitative analysis included the Knee Society Score (KSS), the Western Ontario and McMaster University (WOMAC) index, knee ROM, posterior condylar offset (PCO), and the posterior condylar offset ratio (PCOR).

The methodological quality of the included studies was assessed independently by three authors (M.M., F.N., F.F.); cohort studies were assessed using the Modified Newcastle–Ottawa Quality Assessment [20]. Based on the total score, quality was classified as “low” (0–3), “moderate” (4–6), and “high” (7–9). Randomized controlled trials were assessed with version 2 of the risk of bias tool (RoB2) [21,22], recommended by the Cochrane Collaboration. Discrepancies were resolved by consulting a senior reviewer (GG) [23]. Details of this quality assessment are shown in Table 1.

### 2.3. Data Synthesis

One decimal place accuracy was used to report all data. For the continuous variables, the counts were recorded, and for the categorical variables, the mean, standard deviation, and range were noted. Pooled mean differences (MDs) from functional and radiographic outcomes were analyzed in a meta-analysis. Based on the between-trials heterogeneity as determined by the I^2^ statistics, either random or fixed-effect models were used; specifically, random-effect models were used when significant heterogeneity was observed, unless the between-studies variance (σ2) was low, in which case fixed-effect models were used despite the heterogeneity. For statistical calculations, Review Manager (RevMan 5.3, Cochrane Collaboration, Nordic Cochrane Center, Copenhagen, Denmark) was utilized; a *p* value < 0.05 was deemed significant.

## 3. Results

**Figure 1:**
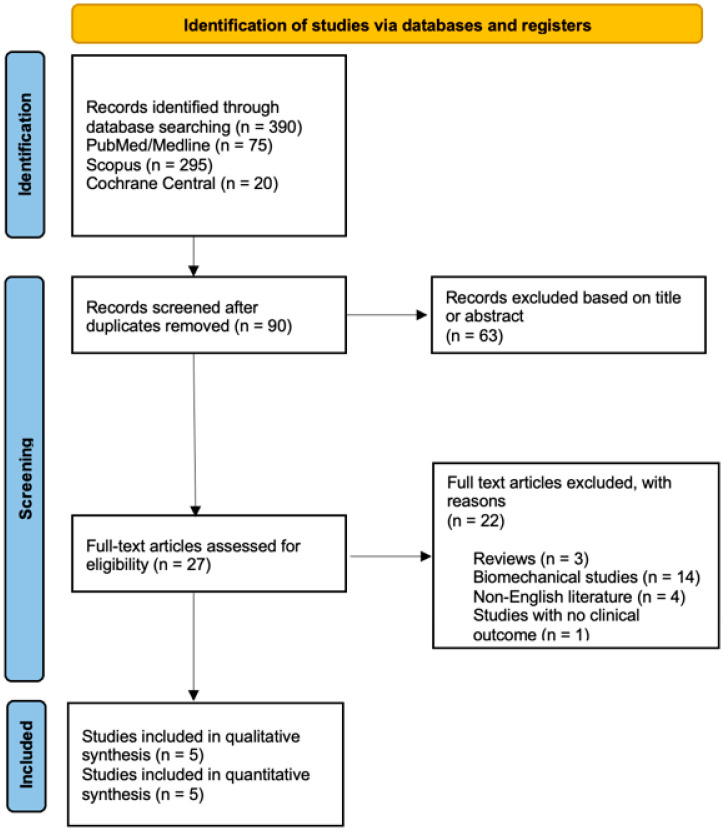
PRISMA (Preferred Reporting Items for Systematic Reviews and Meta-Analyses) flowchart of the study selection criteria.

After the initial search turned up 73 relevant articles, 46 abstracts were screened, and 27 full-text articles were evaluated for eligibility according to our inclusion criteria. This led to the identification of five comparative studies that could be included in the meta-analysis (Figure 1). There were 584 patients in all, 294 of whom did not undergo anterior referencing TKA and 290 of whom did not undergo posterior referencing TKA.

The mean age was 70.4 ± 6.9 years, the mean BMI was 27.6 ± 4.2 kg/m^2^, and the mean follow-up was 40.7 ± 8.1 months. Different types of implant were used for TKA, including posterior stabilized implants Stryker Triathlon (Stryker Orthopaedics, Mahwah, NJ, USA) [24], Zimmer^®^ NexGen^®^ LPS (Zimmer Holdings Inc., Warsaw, IN, USA) [24], Tornier^®^ HLS Noetos^®^ (Tornier Inc., Amsterdam, The Netherlands) [25], U2 Total Knee System (United, Taipei, Taiwan) [11], Legion CR and LPS TKA Systems (Smith & Nephew, Memphis, TN, USA) [10], LOSPA implant (Corentec, Seoul, South Korea) [26], and Vanguard implant (Zimmer Biomet, Warsaw, IN, USA) [26].

### 3.1. Functional Outcomes

**Figure 2:**
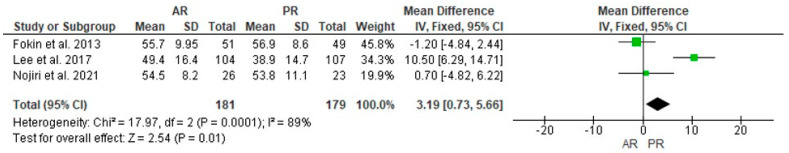
Comparison of preoperative Knee Society Score (KSS) between anterior referencing (AR) and posterior referencing (PR) total knee arthroplasty: forest plot of effect sizes. CI means Confidence Interval. The green square represents the point estimate of each study. The size of the square represents the weight of the study. The black rhombus represents the pooled effect estimates and CI from all studies included in the meta-analysis. References: [10,24,26].

**Figure 3:**
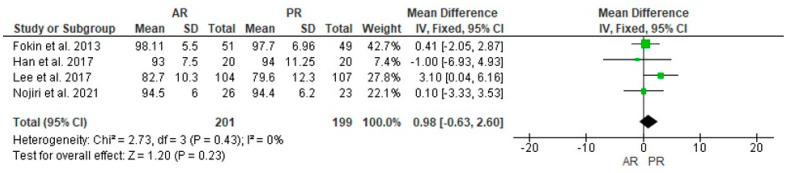
Comparison of postoperative Knee Society Score (KSS) between anterior referencing (AR) and posterior referencing (PR) total knee arthroplasty: forest plot of effect sizes. CI means Confidence Interval. The green square represents the point estimate of each study. The size of the square represents the weight of the study. The black rhombus represents the pooled effect estimates and CI from all studies included in the meta-analysis. References: [10,24,26,27].

Preoperative KSS was determinated in three studies in 181 and 179 cases in the AR and PR groups, respectively, with a statistically significant difference found in favor of the PR group (MD = 3.19, 95% CI [0.73, 5.66], *p* = 0.01) (Figure 2); four studies reported postoperative KSS in 201 and 199 cases in the AR and PR groups, respectively, and no statistically significant difference was found (MD = 0.98, 95% CI [−0.63, 2.60], *p* = 0.23) (Figure 3).

**Figure 4:**
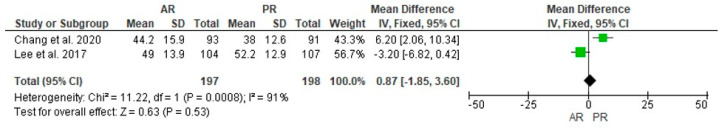
Comparison of preoperative Western Ontario and McMaster University (WOMAC) index between anterior referencing (AR) and posterior referencing (PR) total knee arthroplasty: forest plot of effect sizes. CI means Confidence Interval. The green square represents the point estimate of each study. The size of the square represents the weight of the study. The black rhombus represents the pooled effect estimates and CI from all studies included in the meta-analysis. References: [11,26].

**Figure 5:**
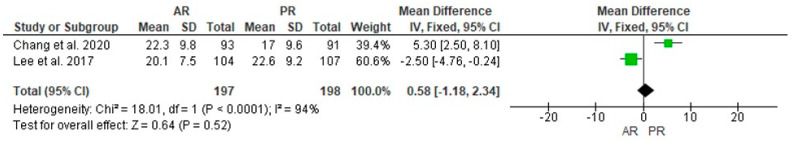
Comparison of postoperative Western Ontario and McMaster University (WOMAC) index between anterior referencing (AR) and posterior referencing (PR) total knee arthroplasty: forest plot of effect sizes. CI means Confidence Interval. The green square represents the point estimate of each study. The size of the square represents the weight of the study. The black rhombus represents the pooled effect estimates and CI from all studies included in the meta-analysis. References: [11,26].

WOMAC was measured preoperatively in two studies in 197 and 198 cases in the AR and PR groups, respectively, and no statistically significant differences were found (MD = 0,87, 95% CI [−1.85, 3.60], *p* = 0.53) (Figure 4); two studies questioned the WOMAC index postoperatively in the same cases and the analysis showed no difference between the two groups (MD = 0.58, 95% CI [−1.18, 2.34], *p* = 0.52) (Figure 5).

**Figure 6:**
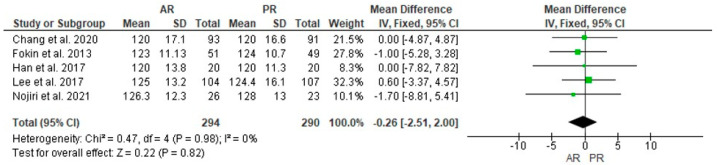
Comparison of preoperative range-of-motion (ROM) between anterior referencing (AR) and posterior referencing (PR) total knee arthroplasty: forest plot of effect sizes. CI means Confidence Interval. The green square represents the point estimate of each study. The size of the square represents the weight of the study. The black rhombus represents the pooled effect estimates and CI from all studies included in the meta-analysis. References: [10,11,24,26,27].

**Figure 7:**
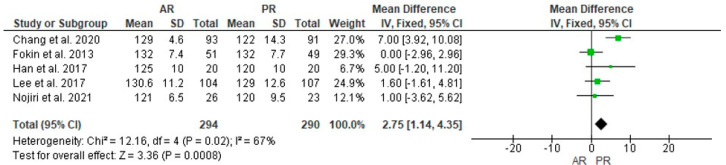
Comparison of postoperative range-of-motion (ROM) between anterior referencing (AR) and posterior referencing (PR) total knee arthroplasty: forest plot of effect sizes. CI means Confidence Interval. The green square represents the point estimate of each study. The size of the square represents the weight of the study. The black rhombus represents the pooled effect estimates and CI from all studies included in the meta-analysis. References: [10,11,24,26,27].

Five studies with 294 and 290 patients in the AR and PR TKA groups, respectively, reported the preoperative ROM, and no statistically significant difference was found (MD = −0.26, 95% CI [−2.51, 2.00], *p* = 0.82) (Figure 6); postoperative ROM was reported in the same cases, and a statistically significant difference was found in favor of the AR group (MD = 2.75, 95% CI [1.14, 4.35], *p* < 0.001) (Figure 7).

### 3.2. Radiographic Outcomes

**Figure 8:**
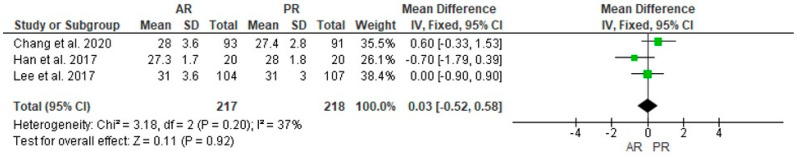
Comparison of posterior condylar offset (PCO) between anterior referencing (AR) and posterior referencing (PR) inTKA: forest plot of effect sizes. CI is Confidence Interval. The green square represents the point estimate of each study. The size of the square represents the weight of the study. The black rhombus represents the pooled effect estimates and CI from all studies included in the meta-analysis. References: [11,26,27].

**Figure 9:**
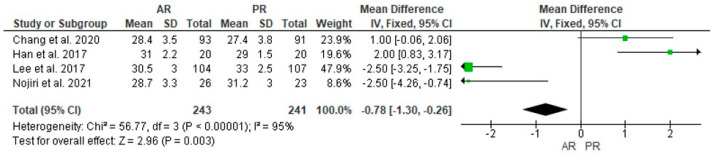
Comparison of posterior condylar offset (PCO) between anterior referencing (AR) and posterior referencing (PR) total knee arthroplasty: forest plot of effect sizes. CI means Confidence Interval. The green square represents the point estimate of each study. The size of the square represents the weight of the study. The black rhombus represents the pooled effect estimates and CI from all studies included in the meta-analysis. References: [10,11,26,27].

Three studies reported preoperative PCO in 217 and 218 cases in the AR and PR TKA groups, respectively, and was not found any statistically significant difference (MD = 0.03, 95% CI [−0.52, 0.58], *p* = 0.92) (Figure 8); four studies described postoperative PCO in 243 and 241 cases in the AR and PR TKA groups, respectively, and a statistically significant difference was found with a higher postoperative PCO in the PR group (MD = −0.78, 95% CI [−1.30, −0.26], *p* = 0.003) (Figure 9).

**Figure 10:**
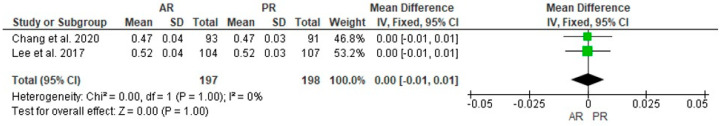
Comparison of posterior condylar offset ratio (PCOR) between anterior referencing (AR) and posterior referencing (PR) total knee arthroplasty: forest plot of effect sizes. CI stands for Confidence Interval. The green square represents the point estimate of each study. The size of the square represents the weight of the study. The black rhombus represents the pooled effect estimates and CI from all studies included in the meta-analysis. References: [11,26].

**Figure 11:**
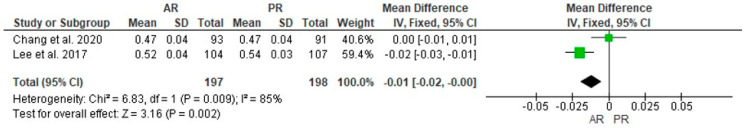
Comparison of posterior condylar offset ratio (PCOR) between anterior referencing (AR) and posterior referencing (PR) total knee arthroplasty: forest plot of effect sizes. CI means Confidence Interval. The green square represents the point estimate of each study. The size of the square represents the weight of the study. The black rhombus represents the pooled effect estimates and CI from all studies included in the meta-analysis. References: [11,26].

PCOR was calculated preoperatively in two studies in 197 and 198 implants positioned with AR and PR TKA groups, respectively, and no statistically significant difference was found (MD = 0.00, 95% CI [−0.01, 0.01], *p* = 1.00) (Figure 10). Postoperatively, PCOR was determinated in two studies in the same cases in the AR and PR TKA groups and a statistically significant difference was found with a higher postoperative PCOR in the PR group (MD = −0.01, 95% CI [−0.02, −0.00], *p* = 0.002) (Figure 11).

## 4. Discussion

The central findings of this study offer a compelling foundation for a more extensive exploration into the intricate dynamics between AR and PR techniques in the realm of TKA. Over the course of a mean follow-up period spanning 40 months, this research has not only provided insightful revelations but also unearthed noteworthy distinctions in outcomes, paving the way for a thorough examination of their implications on critical aspects such as knee function, patient satisfaction, and an array of clinical measures.

The revelation that AR for TKA yielded a significant improvement in knee ROM, juxtaposed with PR showcasing significantly enhanced postoperative imaging scores, marks a pivotal starting point for delving deeper into the multifaceted world of knee arthroplasty. This striking dichotomy in outcomes serves as a catalyst for a more nuanced understanding of the distinct advantages and potential drawbacks associated with each referencing technique. Interestingly, the absence of discernible differences between AR and PR concerning clinical outcome measures, including pain, function, and satisfaction, introduces a layer of complexity to the decision-making process for orthopedic surgeons. This highlights the imperative for a nuanced understanding of the implications associated with each referencing technique, urging a deeper exploration into the subtle intricacies that shape patient outcomes in TKA procedures.

Building upon the current findings, an in-depth examination of KSS and WOMAC scores reveals a consistent narrative. In harmony with prior reports [24,27,28], this study establishes that no significant differences exist between AR and PR groups, reinforcing the notion that both referencing systems can reliably yield satisfactory clinical outcomes. The noteworthy studies conducted by Fokin et al. and Han et al., where patients were randomly assigned to AR or PR techniques, echo this sentiment by reporting no significant differences in intraoperative and clinical outcomes [24,27]. These consistent findings underscore the robustness of both referencing systems and the potential for surgeons to adopt either with confidence, emphasizing the versatility inherent in contemporary orthopedic practices.

However, the glaring scarcity of studies directly comparing the clinical results of anterior and posterior referencing methods serves as a clarion call for future randomized controlled trials. The paucity of a substantial body of evidence comparing these techniques raises fundamental questions about the optimal referencing system for enhancing TKA clinical outcomes. While this study has provided valuable insights, it functions as a catalyst, urging the scientific community to delve deeper into this critical area through well-designed trials that can offer more definitive conclusions and contribute to a more nuanced understanding of the intricate nuances associated with each technique.

The meta-analysis component of this study adds a layer of granularity to the exploration, unraveling a significant difference in postoperative knee ROM between the AR and PR groups. The emphasis on significantly higher knee ROM in the AR group, positioned relative to the anterior femoral condyles, suggests a potential advantage in facilitating more natural knee motion and reducing the risk of posterior impingement [10]. The 2.75-degree improvement in mean difference at a mean 40 months postoperatively underscores the potential clinical significance of this finding. Nevertheless, it is imperative to balance this potential advantage against the acknowledged technical difficulties associated with AR, which may lead to increased bone loss.

Navigating the technical intricacies associated with AR and PR techniques warrants careful consideration. While AR is postulated to offer advantages in terms of knee ROM, the acknowledged technical difficulty and potential for increased bone loss add layers of complexity to the decision-making process. Conversely, PR, perceived as a technique that is easier to perform and may cause less bone loss, comes with its own set of challenges, such as an increased posterior tibial slope and the associated risk of posterior impingement [29,30]. The delicate balance between these considerations places the onus on surgeons to weigh the potential advantages and disadvantages of both referencing methods for optimizing successful TKA implants. This calls for a comprehensive understanding of the surgical nuances associated with each technique, guiding surgeons in making informed decisions tailored to individual patient needs.

This study delves into postoperative imaging scores, particularly PCO and PCOR. The significantly higher mean postoperative PCO and PCOR in the PR group open the door to intriguing discussions. The restoration of PCO in PR, preventing direct impingement on the posterior femoral condyle and thereby enhancing postoperative knee flexion, adds a layer of complexity to the decision-making process. However, the controversy surrounding the correlation between PCO and postoperative knee flexion, as evidenced by previous studies [31,32,33,34], introduces a level of uncertainty. This study appropriately highlights the need for more research to unravel the clinical implications of anatomically matched designed components that permit anatomic referencing, acknowledging the ongoing controversies in this domain. This underscores the dynamic nature of orthopedic research, urging continued exploration to refine and enhance surgical techniques and patient outcomes.

As with any scientific inquiry, this study is not without limitations. The inclusion of a limited number of studies in the meta-analysis, while based on concise and strict inclusion criteria for quantitative analysis, introduces an element of potential bias. However, this methodological choice was made to enable a quantitative analysis rather than a qualitative systematic review. Additionally, the absence of variables concerning differences in patient demographics and the non-evaluation of bone loss may restrict the generalizability of results. The acknowledgment of these limitations positions the study within the broader context of ongoing research, urging future investigations to delve into differences in patient anatomy and the type of implant used. This recognition underscores the iterative nature of scientific inquiry, urging researchers to build upon existing knowledge and refine methodologies to enhance the depth and breadth of understanding in the field of orthopedics.

## 5. Conclusions

In conclusion, the central findings of this study open up avenues for a more comprehensive understanding of the intricate dynamics between AR and PR techniques in Total Knee Arthroplasty. The nuances uncovered, from improved knee ROM in AR to enhanced postoperative imaging scores in PR, invite further exploration into the subtle complexities that shape patient outcomes. As the scientific community navigates these complexities, this study functions not as a finality but as a catalyst, urging continuous research, evidence-based practices, and the relentless pursuit of refining TKA methodologies. It underscores the imperative for a nuanced approach to decision-making in orthopedic surgeries, emphasizing the dynamic interplay of surgical techniques, patient-specific factors, and ongoing advancements in the field.

## Figures and Tables

**Table 1:** Risk of bias summary: review authors’ judgements about each risk of bias item for each included study. Green means low risk of bias; yellow, unclear risk of bias; red, high risk of bias.

	D1	D2	D3	D4	D5	Overall	
Lee	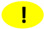	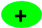	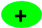	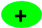	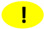	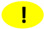	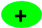
Nojiri	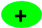	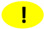	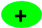	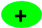	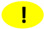	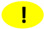	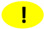
Han	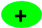	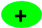	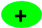	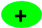	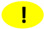	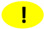	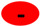
Fokin	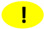	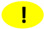	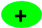	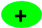	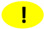	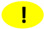	

## Data Availability

The data presented in this study are available on reasonable request from the corresponding author.
